# Supplementary material for: Spatially Resolved Proteomic and Transcriptomic Profiling of Anaplastic Lymphoma Kinase-Rearranged Pulmonary Adenocarcinomas Reveals Key Players in Inter- and Intratumoral Heterogeneity
Source: Int J Mol Sci. 2023 Jul 12;24(14):11369. doi: 10.3390/ijms241411369 (PMC10380216; doi:10.3390/ijms241411369)
Supplement: Supplementary file 1 [file ijms-24-11369-s001.zip › Szeitz et al_IJMS_2023 - Supporting Information.pdf]

Spatially Resolved Proteomic and Transcriptomic Profiling of Anaplastic Lymphoma Kinase-Rearranged Pulmonary Adenocarcinomas Reveals Key Players in Inter- and Intratumoral Heterogeneity

Supporting Information

Supplementary tables

Each Excel file is accompanied by a "README" sheet that provides an introduction to the content of each spreadsheet within the file.

Table S1. Individual ROI annotations and normalized protein/gene expression tables. Sheet "pROI_LFQ_table": A table containing pROI annotations and corresponding normalized LFQ intensities of proteins. **Sheet "pROI_iBAQ_table":** A table containing pROI annotations and corresponding normalized iBAQ intensities of proteins. **Sheet "tROI_table":** A table containing tROI annotations and corresponding normalized gene counts. **Sheet "tROI_table_log2":** A table containing tROI annotations and corresponding normalized and log2-transformed gene counts.

Table S2. MaxQuant and Byonic software settings. Sheet "MaxQuant_Byonic": The most important MaxQuant and Byonic settings.

Table S3. Correlation analyses between pROIs and tROIs. Sheet "Gene_level_corr": Results for gene-level Pearson correlation analyses between pROIs and tROIs. **Sheet "ORA_for_pos_corr_genes":** Pathway enrichment analysis results for genes positively correlated between pROIs and tROIs. **Sheet "Singscore_level_corr":** Results for singscore-level Pearson correlation analyses between pROIs and tROIs.

Table S4. Differential expression analysis results for histopathological data. Sheet "Tumor_vs_NAT_pROIs": Differential expression analysis results for the tumor *vs.* NAT comparison at the pROI level. **Sheet "Tumor_vs_NAT_tROIs":** Differential expression analysis results for the tumor *vs.* NAT comparison at the tROI level. **Sheet "Immune_pROIs":** Differential expression analysis results for the TIL % comparisons at the pROI level. **Sheet "Immune_tROIs":** Differential expression analysis results for the immune score comparisons at the tROI level. **Sheet "Mucin_pROIs":** Differential expression analysis results for the mucin score comparisons at the pROI level. **Sheet "Stroma_pROIs":** Differential expression analysis results for the stroma score comparisons at the pROI level. **Sheet "pGSEA_results":** The detailed pre-ranked GSEA results for all performed differential expression analyses.

Table S5. Proteins and genes contributing to intratumoral homogeneity and heterogeneity. Sheet "pROIs_stable_proteins": List of proteins that were identified as stably expressed in at least one tumor. **Sheet "pROIs_variable_proteins":** List of proteins that were identified as variably expressed in at least one tumor. **Sheet "pROIs_stable_proteins_ORA":** Pathway enrichment analysis for the proteins stably expressed in minimum four tumors. **Sheet "pROIs_variable_proteins_ORA":** Pathway enrichment analysis for the proteins variably expressed in minimum four tumors. **Sheet "tROIs_stable_genes":** List of genes that were identified as stably expressed in at least one tumor. **Sheet "tROIs_variable_genes":** List of genes that were identified as variably expressed in at least one tumor. **Sheet "tROIs_stable_genes_ORA":** Pathway enrichment analysis for the genes stably expressed in minimum four tumors. **Sheet "tROIs_variable_genes_ORA":** Pathway enrichment analysis for the genes variably expressed in minimum four tumors.

Supplementary figures

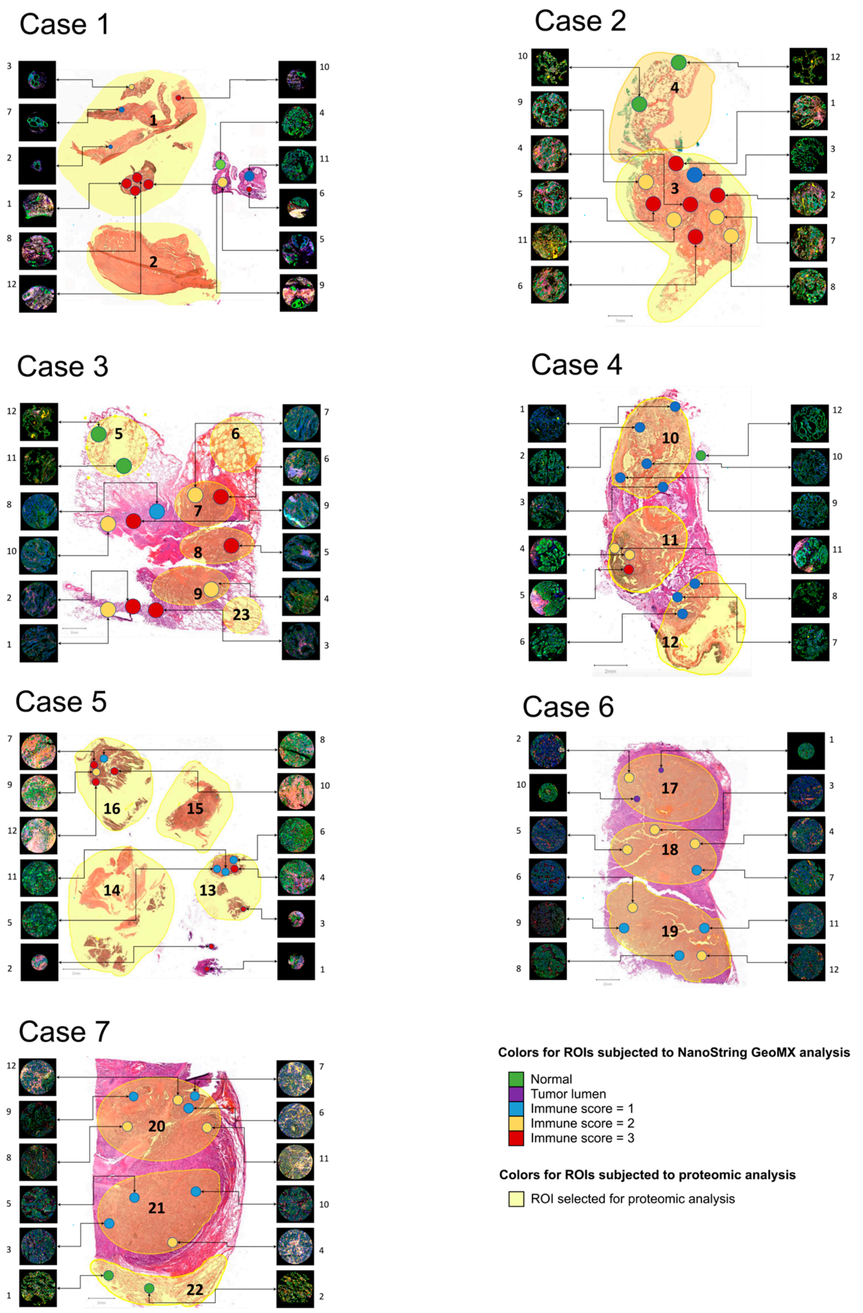

Figure S1. The analyzed ROIs across the seven *ALK*-rearranged pADCs. The smaller ROIs are colored based on immune score or whether the ROI was in a NAT region. The microtome-cut slides were stained with hematoxylin and eosin (HE), and scanned with a Pannoramic Slide scanner. Morphological areas were annotated and immune cell infiltration as percentage was assessed and later grouped into an immune score ranging from 0 to 3. Proteomic analysis followed, based on selection of larger tumor areas and NATs. After fluorescent staining (FS) and digitalization of the adjacent slides, tumor cells, stroma and lymphocytes were specifically revealed, and the regions of tumor cells with various levels of immune score and NATs were selected. These selected ROIs were subsequently subjected to gene expression profiling. HE slides 0,4x magnification, FS inserts 63x magnification.

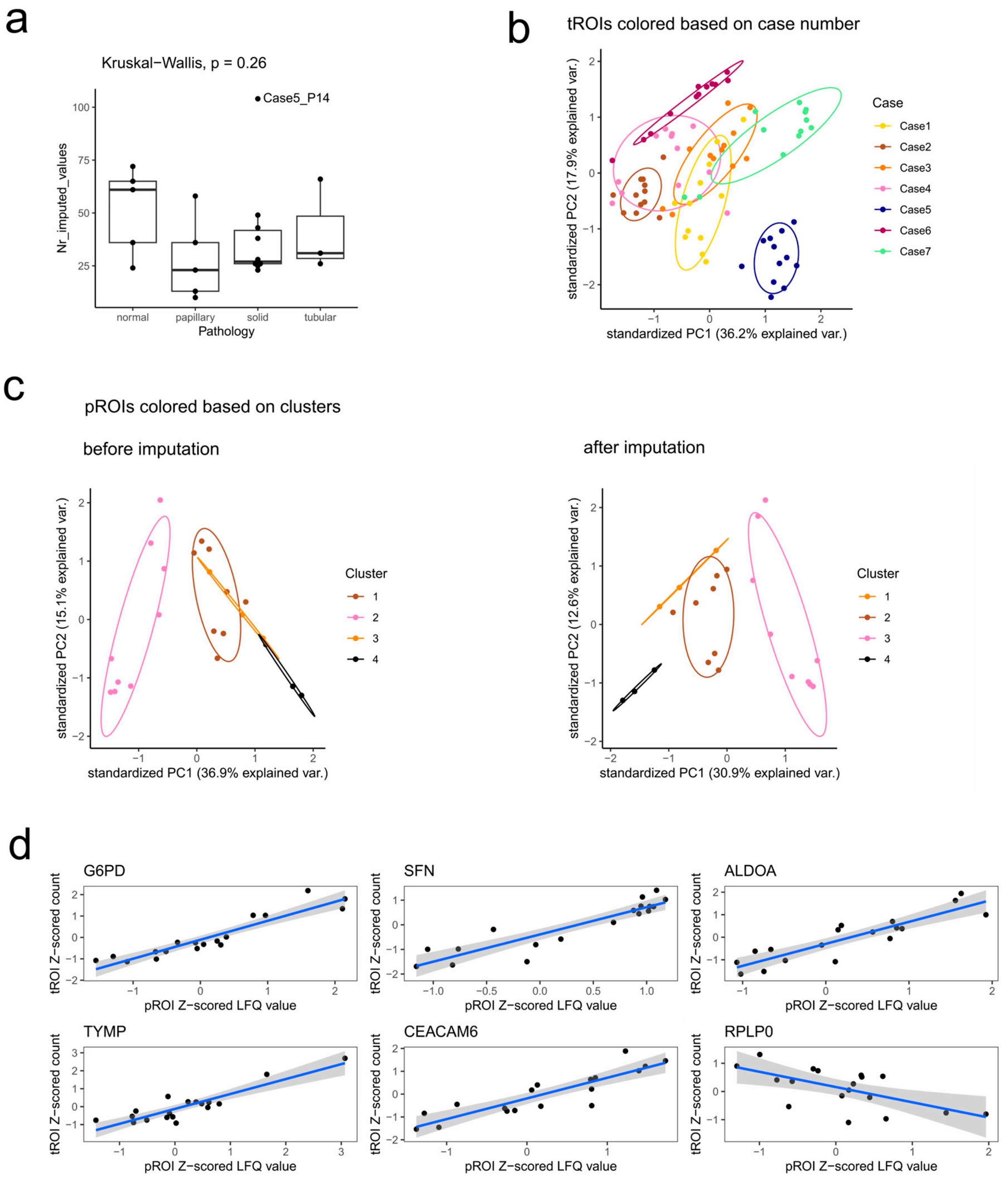

Figure S2. Missing value imputation, PCA plots and the top significantly correlating genes between pROIs and tROIs. (**a**) Number of imputed values across NATs and tumor morphology categories. The pROI with the highest number of imputed values is labeled. (**b**) PCA of tROIs, where samples are colored based on case number. (**c**) PCA of pROIs before (left) and after (right) imputation. Samples are colored based on k-means clustering results. To show PCA plot before imputation, all proteins containing missing values in any of the samples were removed. (**d**) Top positively and negatively correlating genes between pROIs and tROIs.

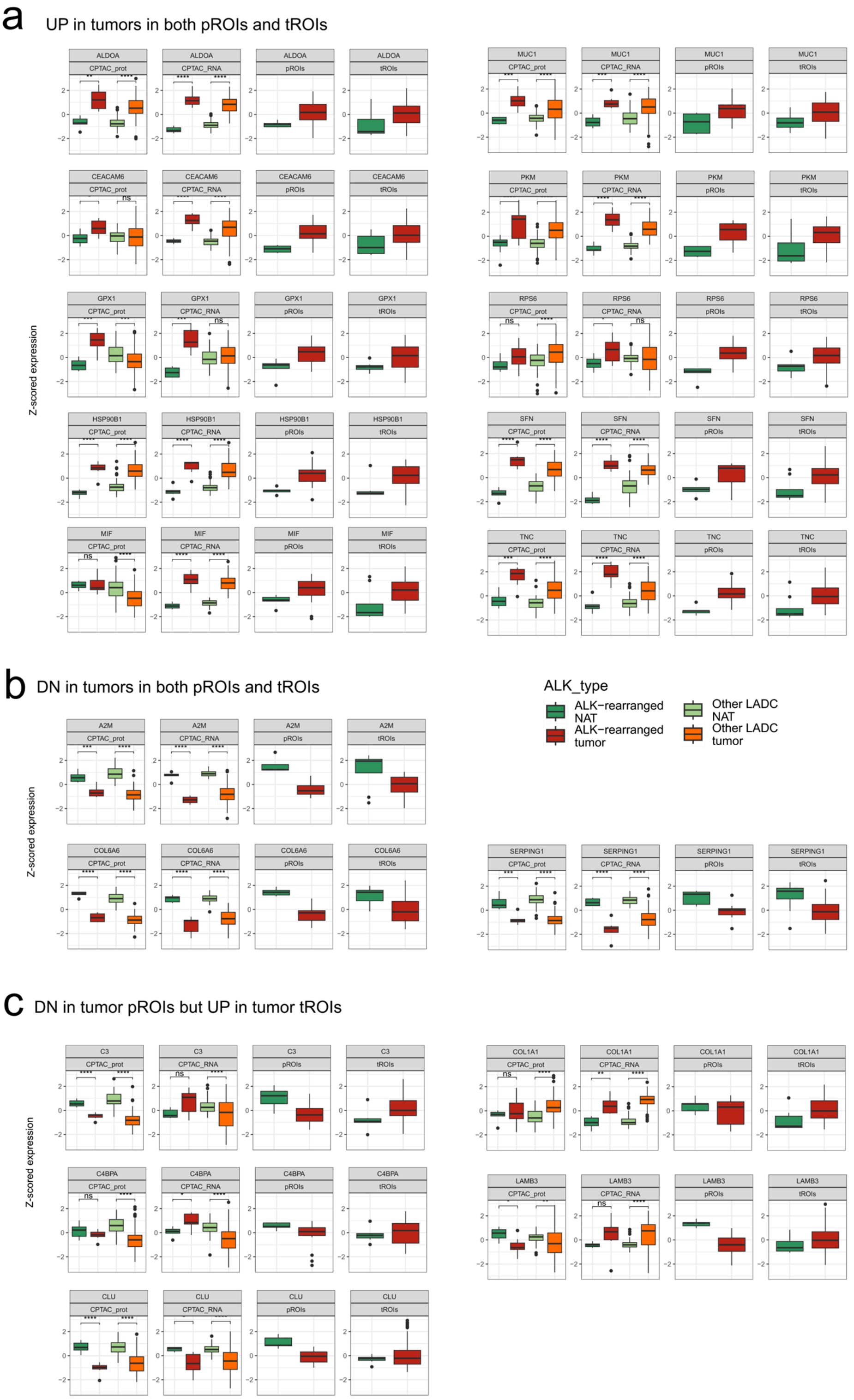

Figure S3. Proteins and genes associated with tissue type in this study, and their expression profile in the CPTAC data. (**a**) Proteins and genes significantly upregulated in tumors compared to NATs in this study. (**b**) Proteins and genes significantly downregulated in tumors compared to NATs in this study. (**c**) Proteins and genes showing opposite tendencies at the pROI and tROI level for the tumor *vs.* NAT comparison in this study. Symbols indicating t-test significance (noted as: ns: $p > 0.05$, *: $p <= 0.05$, **: $p <= 0.01$, ***: $p <= 0.001$) are shown above the boxplots for the CPTAC data.

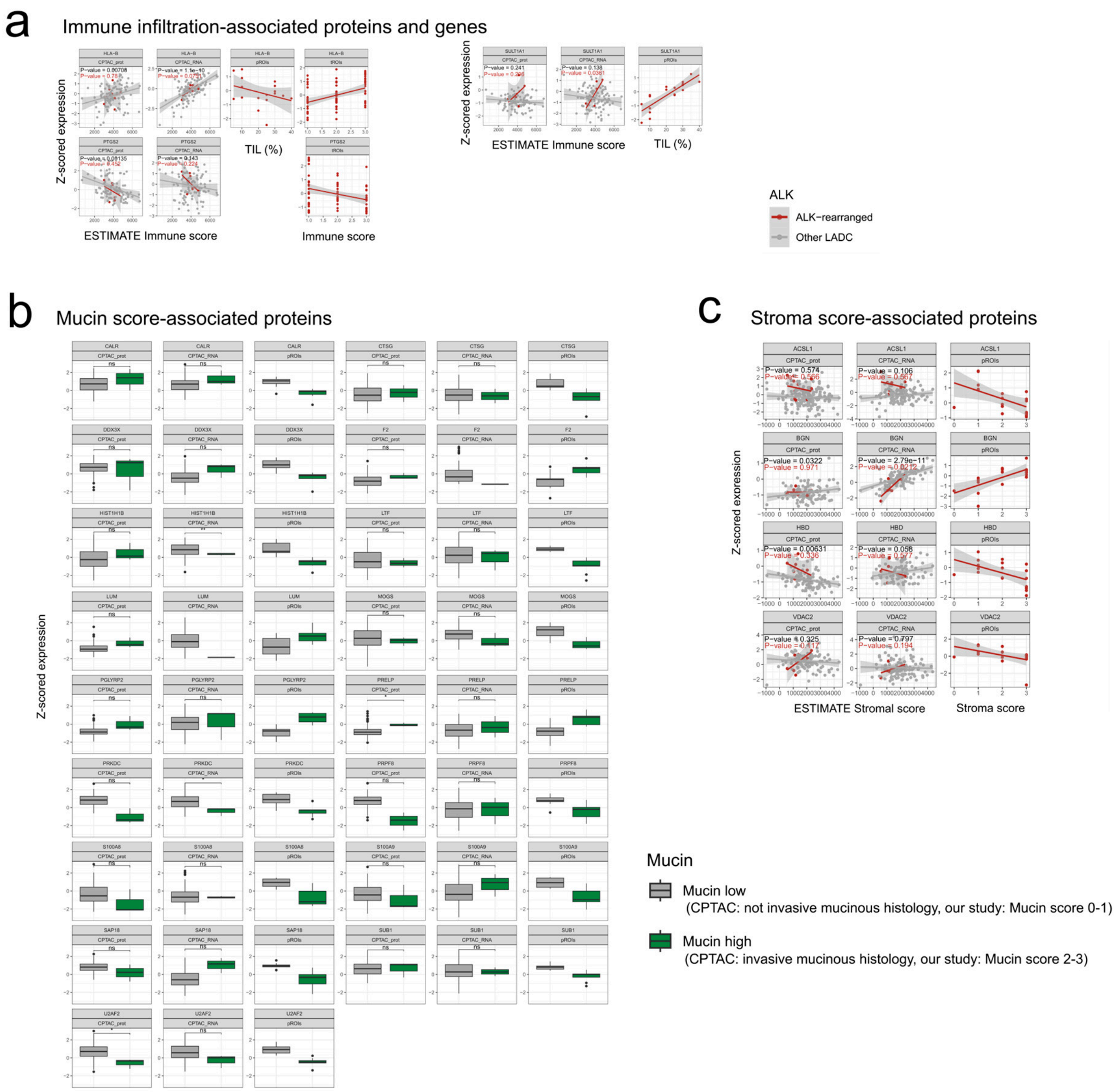

Figure S4. Proteins and genes associated with immune infiltration and stroma/mucin-score differences, and their patterns in the CPTAC data. (**a**) Proteins and genes differentially expressed with immune infiltration in this study. (**b**) Proteins differentially expressed with mucin score in this study. (**c**) Proteins differentially expressed with stroma score in this study. Symbols indicating t-test significance (noted as: ns: $p > 0.05$, *: $p <= 0.05$, **: $p <= 0.01$, ***: $p <= 0.001$) or linear regression p-values are shown above the boxplots/scatter plots for the CPTAC data.

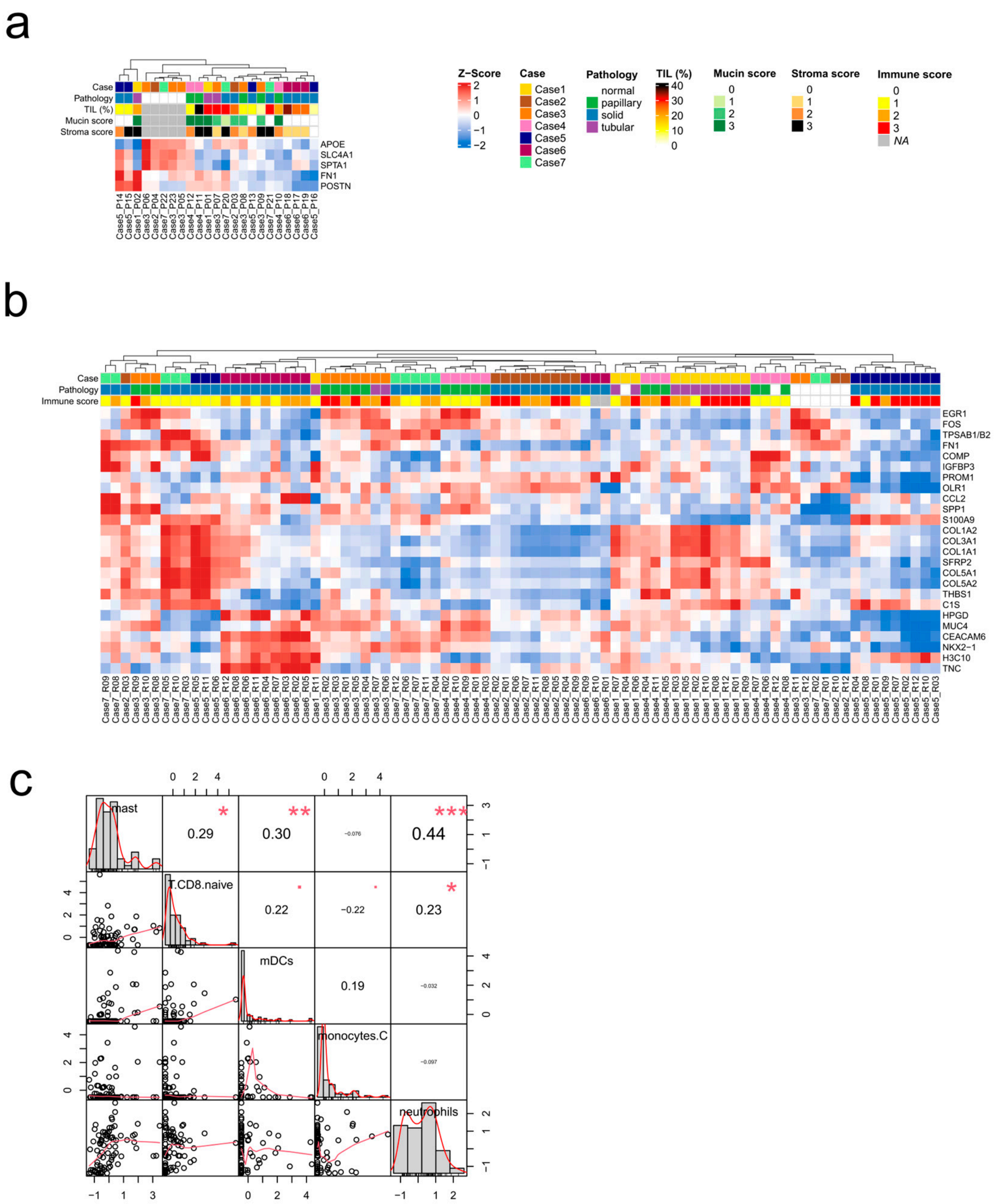

Figure S5. Proteins and genes with the strongest contribution to intratumoral heterogeneity across the seven pADCs with *ALK* rearrangements. (**a**) Proteins that were highly variable within all six tumors that consisted of minimum two tumor regions at the pROI level. (**b**) Genes that were highly variable within all seven tumors at the tROI level. (**c**) Correlation chart displaying the relationship between TME elements forming the most distinct cluster (row cluster 3, see Figure 3).
